# Bioactive Agent Discovery from the Natural Compounds for the Treatment of Type 2 Diabetes Rat Model

**DOI:** 10.3390/molecules25235713

**Published:** 2020-12-03

**Authors:** Shih-Chun Yang, Ching-Yun Hsu, Wei-Ling Chou, Jia-You Fang, Shih-Yi Chuang

**Affiliations:** 1 Department of Cosmetic Science, Providence University, Taichung 43301, Taiwan; yangsc@pu.edu.tw; 2 Department of Nutrition and Health Sciences, Chang Gung University of Science and Technology, Kweishan, Taoyuan 33303, Taiwan; cyhsu@mail.cgust.edu.tw; 3 Research Center for Food and Cosmetic Safety, Chang Gung University of Science and Technology, Kweishan, Taoyuan 33303, Taiwan; fajy@mail.cgu.edu.tw; 4 Research Center for Chinese Herbal Medicine, Chang Gung University of Science and Technology, Kweishan, Taoyuan 33303, Taiwan; 5 Department of Traditional Chinese Medicine, Chang Gung Memorial Hospital, Keelung 20401, Taiwan; chouweiling@gmail.com; 6 Pharmaceutics Laboratory, Graduate Institute of Natural Products, Chang Gung University, Kweishan, Taoyuan 33303, Taiwan; 7 Department of Anesthesiology, Chang Gung Memorial Hospital, Kweishan, Taoyuan 33303, Taiwan

**Keywords:** animal model, drug development, herbal medicine, insulin resistance, Type 2 diabetes mellitus

## Abstract

Diabetes mellitus is a well-known chronic metabolic disease that poses a long-term threat to human health and is characterized by a relative or absolute lack of insulin, resulting in hyperglycemia. Type 2 diabetes mellitus (T2DM) typically affects many metabolic pathways, resulting in β-cell dysfunction, insulin resistance, abnormal blood glucose levels, inflammatory processes, excessive oxidative reactions, and impaired lipid metabolism. It also leads to diabetes-related complications in many organ systems. Antidiabetic drugs have been approved for the treatment of hyperglycemia in T2DM; these are beneficial for glucose metabolism and promote weight loss, but have the risk of side effects, such as nausea or an upset stomach. A wide range of active components, derived from medicinal plants, such as alkaloids, flavonoids, polyphenol, quinones, and terpenoids may act as alternative sources of antidiabetic agents. They are usually attributed to improvements in pancreatic function by increasing insulin secretions or by reducing the intestinal absorption of glucose. Ease of availability, low cost, least undesirable side effects, and powerful pharmacological actions make plant-based preparations the key player of all available treatments. Based on the study of therapeutic reagents in the pathogenesis of humans, we use the appropriate animal models of T2DM to evaluate medicinal plant treatments. Many of the rat models have characteristics similar to those in humans and have the advantages of ease of genetic manipulation, a short breeding span, and access to physiological and invasive testing. In this review, we summarize the pathophysiological status of T2DM rat models and focus on several bioactive compounds from herbal medicine with different functional groups that exhibit therapeutic potential in the T2DM rat models, in turn, may guide future approach in treating diabetes with natural drugs.

## 1. Introduction

**Figure 1:**
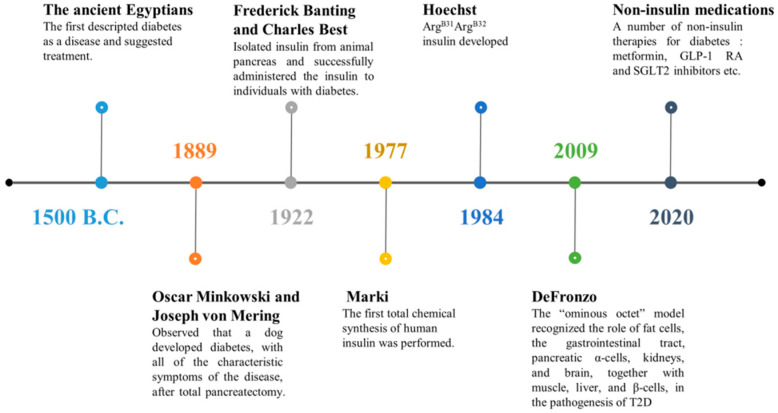
A timeline highlighting represents the development of insulin and T2DM as a therapeutic agent for the treatment of diabetes and the development of modified insulin products with more prolonged times of action.

Type 2 diabetes mellitus (T2DM) is a chronic, complex multisystem disease that causes multiple diabetes-related comorbidities, and requires a multifaceted and personalized approach to treatment. Although the complexity of T2DM is now more exhaustively understood, the scientific community believed that diabetes was a simple disease of the pancreas in the early 19th century. Over the past 30 years, people have acquired a deeper understanding and gained new insights about important contributors to T2DM, including the liver, muscle, kidney, fat cells, brain, α-cells, β-cells, and intestines, as well as various hormones and even systemic inflammation, genetics, and the environment (Figure 1). From an enhanced scientific knowledge of the pathophysiologic progression of T2DM [1,2,3,4,5,6], new treatment options have become possible, thereby, increasing the potential for improving control over this complex disease. However, we need more suitable T2DM animal models for enhanced knowledge on both the pathophysiological progression and potential therapeutic drugs. Furthermore, those animal models must be relevant to the T2DM study, that the characteristics of the animal disease models should mimic the pathophysiology and the inherent history of the disease, or the model should develop T2DM complications with an etiology similar to clinical presentation and the pathophysiology of human disease. Insulin resistance (IR) classically refers to impaired sensitivity to insulin-mediated glucose disposal in muscles, body fat, and liver. Traditional Chinese herbal medicine has been found to play an important role in the T2DM treatment, by attenuating IR and regulating glucose tolerance, and other related mechanisms. In this review, we utilized the T2DM rat models, including genetically spontaneous and experimentally-induced diabetes models, characterized like the clinical manifestation of T2DM by hyperglycemia and IR, which is easily accessible and inexpensive. Furthermore, several bioactive compounds from herbal medicine with different functional groups, such as alkaloids, flavonoids, polyphenols, quinones, and terpenoids, exhibiting therapeutic potential in the T2DM rat models and in turn, may guide our approach in treating diabetes with natural drugs.

## 2. Pathogenesis of T2DM

Diabetes is a common chronic metabolic disease characterized by hyperglycemia due to the deficiency of insulin secretion. Chronic hyperglycemia follows with a variety of complications, including retinopathy, nephropathy, neuropathy, and cardiovascular disorders [7]. The two most common types of diabetes are type 1 diabetes (T1DM) and T2DM. T1DM is generally thought to be caused by immune-related destruction of pancreatic β-cells producing insulin [8]. It is considered an autoimmune disease, and is most common in children and young people. T2DM is associated with genetics, obesity, poor dietary habits, and generally poor nutrition. Unlike T1DM, T2DM is not a simple condition of insufficient insulin secretion. Obesity is one of the main predispositions towards the pathogenesis of T2DM. When nutrient intake is overloaded, the remaining calories from fat are stored as adipose tissue. Nonetheless, the capacity of fat cells to store calories is limited. Too much fat tissue leads to obesity and infiltration of inflammatory cells, resulting in chronic low-grade inflammation, increased lipolysis, and altered secretion of fat hormones (adipokines), including decreased blood adiponectin concentration, all contribute to IR in fat tissue [9,10,11]. This progression leads to disorders related to insufficient or abnormal insulin secretion, which affects the regulation of blood glucose metabolism [12]. Qing He’s group has identified that a hypoxia response in adipose tissue has been reported during obesity. Hypoxia-inducible factor-1 (HIF-1) is activated during hypoxia, resulting in increased expression of c-Jun N-terminal kinase (JNK) and nuclear factor kappa-B kinase (NF-κB) to produce inflammation in adipose tissue [13]. A large number of inflammatory cytokines are released to further exacerbate IR and lipolysis. The aggravation of inflammation further impairs the regulation of peroxisome proliferator-activated receptor (PPAR) and accelerates fat cell death [14,15,16]. With the development of IR, hyperinsulinemia further causes free fatty acid (FFA) to be released from lipoprotein triglycerides hydrolysis [17]. Especially when the capacity of adipose tissue is insufficient, the remaining fat will accumulate in other body tissues, such as the liver, muscle, and even pancreas rather than fatty tissue, through a process called ectopic fat accumulation. For example, the accumulation of fat in striated muscle tissue reduces the glucose uptake of muscle. When fat accumulates in tissues other than adipose tissue, it will further aggravate IR. Therefore, increased FFA flux in IR and adipose tissue forms a vicious circle.

**Figure 2:**
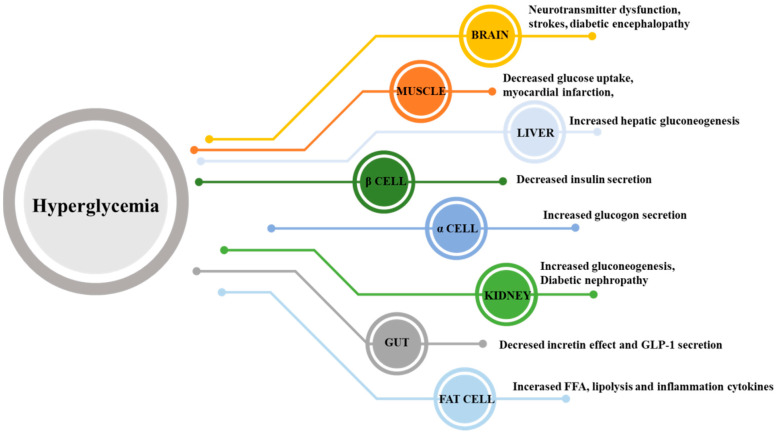
The ominous octet. Multiple defects of organ result from the development of glucose intolerance in T2D. The classical organ systems are targets for which available, including the pancreatic islet, liver, muscle and adipose tissue. The non-classical new organs interventions have been new targeted, have been more focus recently, including the intestine, kidney and brain.

High levels of FFA are released from fat cells into the circulation and accumulate in other organs, thereby, further inducing lipid toxicity and accelerating systemic IR. For example, the accumulation of fat in striated muscle tissue reduces the glucose uptake of muscle. The accumulation of fat in the pancreas will also result in blocking insulin secretion and further cause a rise in blood glucose [17]. Therefore, considering the increased complexity of T2DM mechanism, its progression directly involves different organ systems. In Figure 2, DeFronzo described a systemic approach to the treatment of T2DM and provides eight key targets for therapeutic intervention, including fat cells, gastrointestinal tract, pancreatic α-cells, β-cells, kidneys, and brain, together with skeletal muscle, and liver [6]. Reduced insulin sensitivity in the liver, muscle, and adipose tissue, and progressive dysfunction of pancreatic β-cells lead to impaired insulin secretion, and finally result in hyperglycemia. In patients with T2DM, IR leads to increased lipolysis and increased free fatty acid concentrations in fat cells, resulting in lipotoxicity [18]. Incretin hormone glucagon-like peptide-1 (GLP-1) are gut-derived hormones that can stimulate insulin secretion, and GLP-1 secretion in patients with T2DM is deficient resulting in promoting the process of T2DM [19]. Moreover, sodium-glucose cotransporter 2 (SGLT-2) expression in the kidneys increases excessively, inducing the kidney to continue reabsorbing glucose, rather than excrete it in the urine. This condition is considered to contribute to the maintenance of hyperglycemia in patients with T2DM [20]. Finally, IR also affects the brain and may cause damage of the hypothalamic regions of the brain, associated with appetite regulation. Hypothalamic IR in the brain can contribute to hyperglycemia in T2DM [21]. Overall, this polymorphism model of T2DM provides guidance for therapeutic interventions and also shows that the multisystem dysfunction of T2DM requires a combination of treatments for multiple diseases, rather than a single therapy to target one deficiency.

## 3. The Current Drug Therapy for T2DM

Many antidiabetic drugs have been approved for the treatment of hyperglycemia due to T2DM [12]. Table 1 presents the various classes of available hypoglycemic drugs, based on the DeFronzo’s description of T2DM pathogenesis and their target organs with pathophysiological defects. The classical and non-classical therapeutic target organ systems, include pancreatic islet, liver, muscle and adipose tissue, the intestine, kidney, and the brain. Metformin, as the first-line oral therapy remains the first drug of choice for all T2DM patients. The major mechanism of action, includes a decrease in hepatic gluconeogenesis and an increase in glucose uptake by skeletal muscle [12], reduction of IR via modification of glucose metabolic pathways, and promotion of weight loss. Glucagon-like peptide 1 (GLP-1) receptor agonists (e.g., liraglutide and exenatide) exhibit increased resistance to dipeptidyl peptidase 4 degradation [22,23], and has many clinical benefits, including inhibition of glucagon secretion by the α-cells, stimulation of insulin secretion, and delayed gastric emptying thus promoting weight loss [24]. Moreover, there is a reduced risk of several side effects, including dyslipidemia, hypertension, and endothelial dysfunction [25]. Dipeptidyl peptidase 4 inhibitors (e.g., sitagliptin, vildagliptin, and saxagliptin), which can be taken orally could reduce endogenous GLP-1 degradation. They prolong the circulating half-life of endogenous incretins, thereby providing GLP-1 at physiological levels [22]. Thiazolidinediones (e.g., rosiglitazone and pioglitazone) are PPAR-γ activators that cause an increase in adipokines levels, such as adiponectin, insulin sensitivity by acting on adipose, muscle, and liver tissue to increase glucose uptake and decrease hepatic glucose production [26,27]. Pioglitazone shows a potentially beneficial impact on cardiovascular disease, although it also carries a risk of bladder cancer [27]. The drug may cause side effects, such as fluid retention and edema, weight gain, and increased risk of heart failure. The sodiumglucose co-transporter 2 (SGLT-2) inhibitors are a novel group of compounds that antagonize the glucose transporter, which is responsible for about 90% of glucose reabsorption and is found primarily in the kidney. When this transporter is antagonized, excessive glucose in the renal tubules is not reabsorbed and is excreted in the urine [28], thus, resulting in hyperglycemia reduction. However, the primary side effect of SGLT-2 inhibition is an increase in urinary or genital infections.

## 4. Rat Models of T2D

Thoroughly characterized and clinically relevant animal models also play a vital role, and are promptly needed in understanding the pathogenesis of diabetes. These basic studies can combine the genetic and functional characteristics of diabetes to replace direct drug tests in humans. To further understand the T2DM disorder, many animal models have been developed to demonstrate the pathophysiology and complications of diabetes [29] and to achieve the purpose of managing T2DM with more effective and better therapeutics. Both genetically spontaneous and experimentally induced diabetes models were exist [30]. The symptom patterns induced in these animal models often include obesity, impaired glucose tolerance, IR, and the β-cell models that reflect the human condition in which obesity is closely related to T2DM development (Table 2).

### 4.1. Zucker Fatty Rat and Zucker Diabetic Fatty Rat Models

Zucker fatty (ZF) rat strains, the monogenic models of obesity, are characterized by a deficiency in the leptin receptor that induces hyperphagia and the rats become obese at around 4 weeks of age [36]. These rats are hypertensive, hyperlipidemic, and hyperinsulinemic at around 4–8 weeks of age, and develop advanced IR, glucose intolerance and become completely diabetic. Immunological investigation of the ZF strain exhibits susceptibility to infection [37], T-lymphocytopenia [38], production of immunoglobulins and nitric oxide, and increased expression of tumor necrosis factor-α (TNF-α) and interleukin-1 beta (IL-1β) [39]. The Zucker diabetic fatty (ZDF) rat substrain with a diabetic phenotype is derived by inducing mutations in ZF strains, and they are less obese than the ZF rats. However, these rates exhibit more severe IR due to enhanced apoptosis levels in β-cells, representing a model of obesity-associated diabetes [37].

### 4.2. Otsuka Long Evans Tokushima Fatty Rat

The Otsuka Long Evans Tokushima Fatty (OLETF) rat strain is selectively bred for the null expression of the cholecystokinin-1 receptor in the hypothalamus, resulting in spontaneous hyperphagia, involving obesity and late-onset hyperglycemia at an age of about 20–40 weeks [40,41]. Immunological investigations indicate cellular infiltration and degeneration in pancreatic islets [36]. The late-stage is characterized by hyperplasia, and the islets become fibrotic tissue. These rats also display diabetic nephropathy [42]. This is an appropriate animal model to evaluate both disease progression and skeletal alterations observed in humans [43,44].

### 4.3. Goto-Kakizaki Rat Models

The Goto-Kakizaki (GK) rat, a non-obese model of T2DM, is established by repeated breeding of glucose-intolerant Wistar rats [45]. GK rats have become hyperglycemic at an early age due to an insufficient insulin response resulting in aberrant blood glucose homeostasis [46]. Characteristics of this model, include hyperglycemia, peripheral IR, albuminuria, glomerulosclerosis, tubulointerstitial fibrosis, and development of neuropathy [47]. Retinopathy and neuropathy are also reported to develop late in the life of these animals [48,49]. The islets of GK rats exhibit several biochemical defects, including inflammation, fibrosis [50], decreased energy production, reduced adenylate cyclase activity [51], reduced insulin-stimulated insulin receptor substrate-1 tyrosine phosphorylation [52], and defective regulation of protein phosphatase-1, and -2A and mitogen-activated protein kinase activation in adipocytes [53]. Therefore, precise features of the islets in GK rats resemble those in T2DM patients.

### 4.4. High-Fat Diet/Streptozotocin (HFD/STZ) Rat Model of Diabetes

Streptozotocin (STZ), an antibiotic reagent, causes the production of reactive oxygen species in the β-cells of the pancreas, resulting in β-cell death, and is often used in animal models for the induction of T1DM [54]. In general, this model depends on the toxicity of STZ, which results from the transfer of the methyl-nitrosourea moiety from STZ to the DNA molecule, causing damage and subsequent DNA fragmentation [55]. STZ doses of 65~70 mg/kg are often used to induce T1DM in rats. However, Reed et al. [56] reported a new rat model of T2DM, known as the high-fat diet (HFD)/STZ rat. This model is referred to as a T2DM model, due to similar prediabetes and/or IR symptoms and hypoinsulinemia in patients [56]. Feeding Sprague-Dawley rats with a 40% kcal fat diet for 2 weeks, and subsequent intravenous injection with STZ further establishes the HFD/STZ rat model of T2DM relevant to the human condition [56,57,58]. The HFD/STZ rat models mimic the state of obesity, IR, and/or glucose intolerance in prediabetes conditions [58]. HFD/STZ rats have more pronounced dyslipidemia, glucose intolerance, hyperglycemia, and low levels of circulating adiponectin [59], similar to the metabolic profile of T2DM in humans. Recently, a high fat and sugar diet (HFSD) with the administration of STZ was suggested to establish a T2DM rat model, and it has been reported to be a better, and true mechanism of T2DM pathogenesis given the numerous alterations of protein expression in the INSR/PI3K/AKT pathway and levels of IL-6 and TNF-α [34,60]. No single animal model appears to encompass all of these characteristics, but many exhibit nearly similar characteristics of one or more aspects of T2DM in humans.

## 5. Anti-T2DM Drug Discovery Using T2DM Rat Models

**Figure 3:**
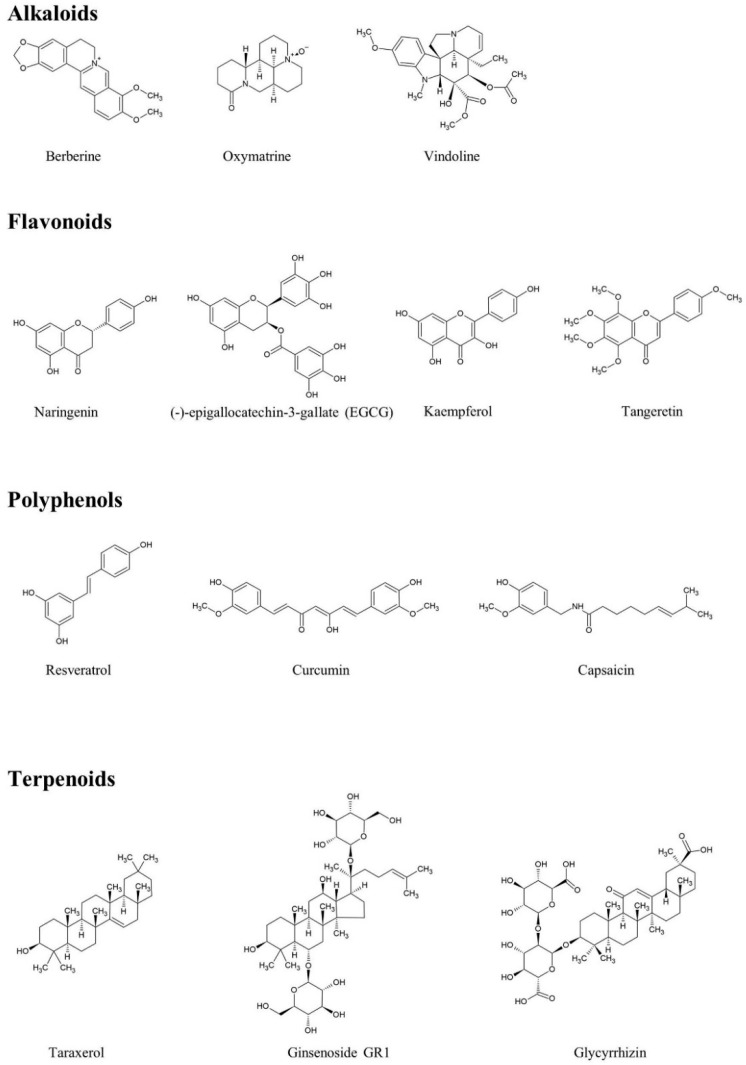
The chemical structures of anti-T2DM compounds introduced in this review.

PubMed database published literature between April 2014 and April 2020 on T2DM rat models that had been treated with herbal medicine compounds were reviewed. For the search, the following combinations of terms were used as keywords: “herbal,” “drug,” “natural compound,” and “insulin resistance” or “T2DM rat models.” Herein, we have summarized the mechanisms of several representative bioactive components in T2DM rat models, including alkaloids, flavonoids, polyphenols, quinones, and terpenoids (Figure 3) used in the treatment of T2DM rat models, and which may help to provide valuable information on the application of pure herbal medicine compounds (Table 3).

### 5.1. Alkaloids

Alkaloids are a class of naturally-occurring compounds, derived from natural sources, such as plants, animals, bacteria, and fungi. They have a wide range of functions, including anti-spasmodic, anti-arrhythmic, anti-malarial, anticancer, antibacterial, and anti-hyperglycemic activity [75]. Various alkaloids, including berberine, oxymatrine, and vindoline are potentially effective against different diabetic models [76]. Berberine is the primary active component of *Rhizoma coptidis* and exhibits antidiabetic properties and hypoglycemic effects. Berberine has been shown to increase insulin and decrease HbA1c, cholesterol (TC), and total glucose (TG) blood levels in the ZDF rat model [61]. Berberine significantly attenuates axonopathy, and also restores PI3K/Akt/GSK3β signaling pathway in HFD/STZ rats. Oxymatrine has been demonstrated to increase serum insulin, liver and muscle glycogen and decrease fasting blood glucose, GLP-1, TC, TG, and muscle glucose transporter-4 levels in HFD/STZ rats [63]. Recently, vindoline, an indole alkaloid from the *Catharanthus roseus* plant, was reported to protect diabetic hepatic tissue from injury via antioxidant, anti-inflammatory, and anti-hypertriglyceridemia activities in a T2DM rat model [64]. Administration of vindoline in an HFD/STZ rat model significantly reduced fasting blood glucose, serum alanine transferase, aspartate aminotransferase, and alkaline phosphatase levels when compared to the diabetic controls [77]. Vindoline also stimulates the activity of superoxide dismutase and catalase and decreases the levels of TNF-α and IL-6. Histopathological findings show that vindoline improves the functions of both hepatic and pancreatic tissues in vivo.

### 5.2. Flavonoids

Flavonoids are bioactive compounds, found in flowers, nuts, fruits, and some vegetables; several investigators have focused on the use of flavonoids and related compounds for antidiabetic properties [77]. Some recent studies have suggested that flavonoid compounds including naringenin, (-)-epigallocatechin-3-gallate (EGCG), rutin, and kaempferol, among other flavonoids, may improve and stabilize the secretion of insulin from pancreatic β-cells. In diabetic animal models, flavonoids typically lead to reduced aldose reductase, regeneration of pancreatic β-cells, and increased insulin release. According to their biological properties, polyphenols may be useful nutraceuticals and supplementary treatments, and are involved in the regulation of carbohydrate and lipid metabolism, amelioration of hyperglycemia, dyslipidemia, and IR, and alleviate oxidative stress and inflammatory signaling pathways [78,79]. Naringin, a major flavanone glycoside obtained from grapefruit, was found to reduce blood glucose and IR index, glycosylated hemoglobin, inflammatory cytokines, and increase the levels of serum insulin, and glutathione in the antioxidant defense system in diabetic rat models [65,80]. Successful uptake of EGCG, a flavonoid-derived from green tea, was reported to improve mitochondrial function and autophagy in the hearts of GK rats with myocardial mitochondrial deficiency and oxidative stress [66]. Rutin is a flavonoid glycoside from flowers and fruits as a major source. In HFD/STZ rats, rutin treated by orally ameliorates the levels of TG and blood glucose, oxidative stress, TNF-α and IL-6 production, and cellular apoptosis pathways [81,82]. Another study found that kaempferol treatment may enhance insulin sensitivity and deterioration of IR in diabetic rats; the possible mechanisms may be the down-regulation of the IKKβ/NF-κB signal and subsequent inhibition of TNF-α and IL-6 production [67].

### 5.3. Polyphenols

Natural polyphenols in the plant kingdom are classified according to the number of phenol rings and structural elements that bind these rings, and include the common polyphenols such as resveratrol, curcumin, and capsaicin. Polyphenols have unique physical, chemical, and biological (metabolic and therapeutic) characteristics, based on the number of aromatic rings and functional groups in the phenol structure [83]. Polyphenols are also the most abundant antioxidants in the human diet. Increasing evidence indicates that various dietary polyphenols might prevent diabetes [78]. Resveratrol is a natural polyphenol widely found in grapes and blueberries [84]. The oral administration of resveratrol in STZ/nicotinamide-induced rats decreased the fasting blood glucose and HbA1c levels and increased the antioxidant activity of superoxide dismutase, catalase, GSH, and glutathione peroxidase. Resveratrol has an important role in controlling hyperglycemia and plasma insulin levels and demonstrates increased expression of PPAR-γ and FALDH in rat adipose tissue [69]. Curcumin is an acidic polyphenolic substance, with multiple physiological and pharmacological activities and remarkably low toxicity [85]. In accordance to some pharmacologic studies, curcumin exhibits neuroprotective effects, anti-hyperlipidemia, and hypolipidemic effects, and improves oxidative stress and inflammation [85,86,87,88]. Pharmacological evidence also indicates that curcumin significantly affects improvement in IR [89] and decrease in blood lipids, inflammatory cytokines [90], and plasma resistin levels [90] in diabetes animal models. In the T2DM rat models, curcumin plays an important role in decreasing fasting blood glucose, inhibiting inflammation, and reducing the apoptosis index in pancreatic islet β-cells in HFD/STZ rats. As part of the underlying mechanism, curcumin reduces TNF-α, IL-1β, IL-6, caspase-3, and Bax levels by blocking the phosphorylation of JNK and NF-κB protein signaling pathway [70]. Clinical characteristics of T2DM reveal a high-risk associated with Alzheimer’s disease due to impaired insulin signaling pathways in brain tissue. Capsaicin is the major pungent compound obtained from hot chili peppers and is a highly selective agonist for the transient receptor potential vanilloid 1 (TRPV1), which was found to ameliorate IR [91]. TRPV1, a Ca2^+^-permeable nonselective cation channel, is expressed mainly in dorsal root ganglion cells and primary sensory afferents in the brains of humans and rats [92]. TRPV1 has a potential therapeutic value for obesity and diabetes [93]. Xu, et al. [71] demonstrated that dietary capsaicin reduced the risk of Alzheimer’s disease in an HFD/STZ rat model. They found that rats receiving dietary capsaicin had a significant decrease in the levels of phosphorylation of AD-associated tau protein at special sites (serine 199, 202, and 396 in the hippocampus) compared with that in T2DM rats. The dietary capsaicin group increased PI3K/AKT and decreased GSK-3β activity, which was also observed in the hippocampus compared with that in T2DM control rats that did not receive capsaicin, indicating that capsaicin inhibited the phosphorylation of tau protein by increasing the PI3K/AKT and inhibiting GSK-3β activity. Dietary capsaicin may have potential use in the prevention of Alzheimer’s disease in T2DM.

### 5.4. Quinones

Quiniones, a class of aromatic dicarbonyl compounds, are found predominantly in flowering plants, and fungi. In nature, quinone is biochemically involved in respiration and photosynthesis, and plays a vital role in electron transport, serving as electron carriers in redox reactions for energy transduction, and storage [94]. Anthraquinones are the largest class of naturally occurring quinones such as rhein, purpurin, and chrysophanol. Rhein is a lipophilic anthraquinone extensively found in medicinal herbs *Rheum palmatum* L. and has many pharmacological effects in protecting against liver and kidney damage, inflammation, excess oxidative reactions, and microbial infections. These pharmacological effects are used to treat hepatic disease [95], diabetes [96], atherosclerosis [97], and cancer [98]. However, many research reports demonstrate that sirtuin 1 (SIRT1) may play a vital role in the control of glucose homeostasis by regulating insulin secretion [99], down-regulating inflammation, improvement of IR [100], controlling fatty acid oxidation and mitochondrial biogenesis, and regulating hepatic glucose production [101]. Gerhart-Hines, et al. [101] indicated that the expression of SIRT1 was reduced in diabetic rats. However, the effect of rhein was to increase SIRT1 expression in HFD/STZ-induced diabetic rats, which improved IR and dyslipidemia. Furthermore, rhein dramatically decreased the levels of fasting plasma glucose, fasting insulin, homeostasis model assessment-insulin resistance index (HOMA-IR), TG, and TC, while renal tissues were significantly improved compared with those in diabetic rats that did not receive rhein. Chrysophanol is an anthraquinone isolated from *Rheum rhabarbarum* belonging to the *Polygonaceae* family. To date, it is known to exhibit several pharmacological effects, including anti-diabetes [102], anti-inflammatory [103], and anti-cancer activity [104]. Chrysophanol targets significantly decrease blood lipids, serum insulin levels in diabetes, and reduces inflammatory cytokines, myocardial enzymes creatine kinase (CK) and lactate dehydrogenase (LDH), and increases SIRT1 protein expression. Chrysophanol also significantly ameliorates the cardiac pathological changes in diabetic animal models. Molecular dynamics studies suggest that chrysophanol inhibits activation of the *High Mobility Group Box 1* (HMGB1)/NF-κB pathway [105], increases GLUT 4 in muscle, induces phosphorylation of insulin receptor substrate-1, and docks well in the active site of DPP-IV, supporting its use as a significant DPP-IV inhibitor [104]. Chrysophanol is a generally used herb in traditional Chinese medicine for improving obesity. A study revealed the anti-obesity effects of chrysophanol by using HFD-induced rat models [106]. Chrysophanol dramatically decreased lipid accumulation in hepatocytes and decreased bodyweight, blood glucose, and blood levels of TG compared to those in HFD rats not receiving chrysophanol. In addition, the herbal compound reduced IL-6 and IL-1β levels and increased IL-10 expression to improve HFD-induced inflammation. The expression of lipolytic genes increased and those of lipogenic genes decreased in HFD rats treated with chrysophanol. Chrysophanol probably benefits from the activation of AMP-activated protein kinase (AMPK)/SIRT1, which leads to the down-regulation of sterol regulatory element-binding protein-1 [106].

### 5.5. Terpenoids

Persistent hyperglycemia causes the activation of protein kinase C (PKC) and the NF-κB signaling pathway associated with the production of IL-1β, IL-6, and TNF-α, revealing that inflammation plays a pivotal role in the development and progression of diabetic nephropathy (DN) [107,108]. Damage originating from the pathogenesis of DN, includes renal inflammation, accumulation of serum creatinine, urea, and uric acid, and release of urinary albumin [109]. Khanra et al. [72] showed that taraxerol, a pentacyclic triterpene from the leaf extract of the plant *Abroma augusta* L., exhibits protective effects against DN via the reduction in the secretion of proinflammatory cytokines, regulation of the serum lipid profile and blood glycemic status, and restoration of the renal physiologic function in T2DM rats. Taraxerol also stimulates the IRS1/PI3K/AKT/AMPK/GLUT4/GSK3β signaling pathways to mediate hyperglycemia and inhibits the PKC/NF-κB signaling pathway to improve inflammatory effects in T2DM rats [72]. Ginsenoside is the major bioactive compound in ginseng, which shows the therapeutic effects of ginsenoside in a diabetic GK rat model, wherein, ginsenoside ameliorated diabetic progression, including the levels of blood glucose, body weight, and Morris correlation index. The possible mechanism involved is inhibition of SOD, malondialdehyde (MDA), and inflammatory cytokines (IL-1β, and IL-6, and TNF-α) [73]. Glycyrrhizin, a glycol-conjugated triterpene from *Glycyrrhiza glabra*, demonstrated anti-inflammatory properties and was shown to inhibit the cytokine activity of HMGB1. A recent study showed that the inhibition of HMGB1 by glycyrrhizin is an effective strategy for reducing kidney inflammation in a ZDF animal model [74]. The kidney cortex of ZDF rats showed an increase in toll-like receptor 4, phospho-p38 MAPK, and IL-1β expression, as well as an increase in macrophages compared to those in controls, and plays a major part in renal dysfunction. Furthermore, glycyrrhizin treatment blocked HMGB1-activated toll-like receptor 4 downstream signaling pathways, which may in turn block the transcriptional expression of the pro-inflammatory cytokines. Glycyrrhizin treatment also ameliorates the expression of macrophages and cell adhesion molecules, and provides protection against hyperglycemia-induced glomerular damage.

## 6. Conclusions

T2DM is a well-known common metabolic disease that is a risk to human health over the long term. Progression of T2DM from prediabetic state to overt diabetes and the development of complications occurs over many years. The assessment of interventions also takes years and requires large resources. The use of the appropriate animal model, based on diabetic syndromes, can provide substantial data on the pathophysiological mechanisms of T2DM in humans. While, no single animal model has been able to address all these characteristics, many animal models can provide very similar characteristics of one or more aspects of human T2DM. In this review article, we focused on the pathophysiological status of T2DM rat models and several bioactive compounds from herbal medicine with different functional groups, which exhibit therapeutic potential for the T2DM rat models, at the same time, guiding our approach to the treatment of diabetes with natural drugs. Many risk factors and pathogenic processes in T2DM have been verified, including hyperglycemia, IR, lipid accumulation, excessive inflammation, oxidative stress, and adipokines, all of which are critically important in treating the disease. The diabetic rat models are considered to play an important role in presenting the pathogenesis of human T2DM and its complications, despite all the other limitations they offer. The diabetic rat models are essential for investigating and developing novel drugs for diabetes and its complications. The occurrence and prevalence of T2DM can be prevented by utilizing appropriate natural compounds derived from plant-based medicines. In this review, we suggested that these natural compounds can be used as drugs or dietary supplements to help prevent and treat T2DM. However, many questions still remain. Many natural compounds mentioned in our report have effects on hypoglycemia against T2DM, however, a notable side effect is that too much decrease in glycemia will cause patients to enter the hypoglycemic state.

## Figures and Tables

**Table 1:** The pathophysiological defects of organs were targeted by various classes of available hypoglycemic drugs.

Organs	Class	Mechanism of Action	Side Effects
α-cell	GLP-1RA (incretin mimetic drugs: exenatide, liraglutide, albiglutide), DPP-4 inhibitors (saxagliptin, sitagliptin)	Stimulation of the GLP-1 receptor, inhibition of GLP-1 degradation	Nausea, gastrointestinal complaints
β-cell	GLP-1RA (incretin mimetic drugs: exenatide, liraglutide, albiglutide), Thiazolidinediones (pioglitazone)	Stimulation of the GLP-1 receptor, reduction of IR and increase transcription of adipokines	Nausea, weight gain
Brain	GLP-1RA (incretin mimetic drugs: exenatide, liraglutide, albiglutide)	Stimulation of the GLP-1 receptor	Nausea,
Fat cell	Thiazolidinediones (pioglitazone)	Reduction of IR and increase transcription of adipokines	Weight gain
Gut	GLP-1RA (incretin mimetic drugs: exenatide, liraglutide, albiglutide)	Stimulation of the GLP-1 receptor	Nausea,
Liver	GLP-1RA (incretin mimetic drugs: exenatide, liraglutide, albiglutide), Thiazolidinediones (pioglitazone), Biguanide (metformin)	Stimulation of the GLP-1 receptor, reduction of IR and increase transcription of adipokines, enhanction the effect of insulin	Nausea, weight gain, lactic acidosis
Muscle	GLP-1RA (incretin mimetic drugs: exenatide, liraglutide, albiglutide), Thiazolidinediones (pioglitazone)	Stimulation of the GLP-1 receptor, reduction of IR and increase transcription of adipokines	Nausea, weight gain
Kidney	SGLT-2 inhibitors (canagliflozin, dapagliflozin, empagliflozin)	Inhibition of SGLT-2 in the kidney	Diabetic ketoacidosis

**Table 2:** Summary of rat models of type 2 diabetes mellitus.

Type of Models	Abnormality	Ref.
**Obese models (monogenic)**		
Zucker fatty (ZF) rats	Obese Hyperinsulinemia, hyperlipidemia, and hypertension IR and glucose intolerance Susceptibility to infection Decrease in T cell number Increase in macrophage number Augmentation of immunoglobulins and proinflammatory cytokines production	[31,32]
**Obese models (polygenic)**		
OLETF rat	Obese Spontaneously hyperplasia Cellular infiltration and degradation Diabetic nephropathy	[32,33]
**Non-obese models**		
Goto-Kakizaki (GK) rat	A decreased β-cell mass Liver and skeletal muscle IR Attenuation of phagocytic activity of macrophages Augmentation of natural IgM production Increased in the T cell ratios in the white blood and decreased B cells.	[32,33]
**Induced obesity**		
HFD/STZ rat	Dysfunction in β-cells IR and hypoinsulinemia Hyperglycemia Increased INSR/PI3K/AKT pathway and decreased levels of IL-6 and TNF-α. Low level of circulating adiponectin	[34,35]

**Table 3:** Natural bioactive compounds for the treatment of rat models of T2DM.

Structures	Treatment	Model	Improvement Effect	Ref.
**Alkaloids**				
Berberine	Oral	ZDF rats, HFD/STZ-induced rats	Increased insulin and decreased levels of HbA1c, TC, and TG Attenuated axonopathy	[61,62]
Oxymatrine	Oral	HFD/STZ-induced rats	Increased serum insulin and GLP-1, TC, TG, and GLUT-4 content	[63]
Vindoline	Oral	HFD/STZ-induced rats	Reduced fasting blood glucose, serum alanine transferase, aspartate aminotransferase, alkaline phosphatase, and levels of TNF-α and IL-6	[64]
**Flavonoids**				
Naringenin	Intragastric	HFD/STZ-induced rats	Decreased blood glucose and IR index, and improved antioxidation	[65]
(-)-epigallocatechin-3-gallate (EGCG)	Oral	Goto-Kakizaki rats	Improved mitochondrial function and autophagy in the heart of GK rats	[66]
Kaempferol	Intragastric	HFD/STZ-induced rats	Attenuated IR effect and inflammatory signal through inhibition of NF-kB and downstream cytokine production	[67]
Tangeretin	Oral	STZ-induced rats	Reduced plasma glucose, increased in the levels of insulin and hemoglobin and modulates the activities of hepatic enzymes	[68]
**Polyphenols**				
Resveratrol	Oral	STZ-nicotinamide-induced rats	Reduction in blood glucose and HbA1c levels Increased antioxidants activities of SOD, CAT, GSH, GPx, and *PPAR-γ* and *FALDH* gene	[69]
Curcumin	Intraperitoneal	HFD/STZ-induced rats	Decreased fasting blood glucose, the pancreatic tissue destruction and apoptosis index, the expression of IL-1β, IL-6, TNF-α Block the phosphorylation of JNK and NF-κB protein to inhibit this signaling pathway	[70]
Capsaicin	Oral	HFD/STZ- induced rats	A TRPV1 agonist Decreased phosphorylation of tau protein Increased activity of PI3K/AKT and decrease activity of GSK-3β	[71]
**Terpenoids**				
Taraxerol	Oral	HFD/STZ-induced rats	Inhibition of hypoglycemic, Insulin-sensitizing and inflammatory effects Activation of IRS1/PI3K/AKT/AMPK/GLUT4/GSK3b and inhibition of PKC/NF-κB	[72]
Ginsenoside	Intragastric	Goto-Kakizaki rats	Improvement of the blood glucose, body weight Inhibition of brain oxidative/nitrosative damage and IL-1β, IL-6, and TNF-α production	[73]
Glycyrrhizin	Intraperitoneal	ZDF rats	A HMGB1inhibitor. Reduced kidney inflammation Blocked TLR4 signaling pathways Blocked the pro-inflammatory cytokines Ameliorate dmacrophage and cell adhesion molecules, Against glomerular damage	[74]
